# Self-Management for Knee Osteoarthritis: A Systematic Review and Meta-Analysis of Randomized Controlled Trials

**DOI:** 10.1155/2022/2681240

**Published:** 2022-03-02

**Authors:** Zugui Wu, Rui Zhou, Yue Zhu, Ziquan Zeng, Zixuan Ye, Zhenbang Wang, Wengang Liu, Xuemeng Xu

**Affiliations:** ^1^The Fifth Clinical Medical College, Guangzhou University of Chinese Medicine, Guangzhou 510405, China; ^2^Guangzhou University of Chinese Medicine, Guangzhou 510006, China; ^3^Baishui Health Center, Qujing 655335, China; ^4^Guangdong Second Traditional Chinese Medicine Hospital, Guangzhou 510405, China; ^5^Qujing Hospital of Traditional Chinese Medicine, Qujing 655000, China

## Abstract

**Background:**

Knee osteoarthritis (KOA) is a high incidence chronic joint disease that seriously affects patients' quality of life, and current treatment methods have limited efficacy. Self-management may be an effective strategy for KOA, and clinicians have been showing increased interest recently. However, the effectiveness of self-management for KOA remains controversial.

**Purpose:**

This study aims to systematically evaluate the effectiveness of self-management for KOA.

**Methods:**

We screened articles published in MEDLINE, Cochrane Library, EMBASE, and Web of Science until September 17, 2021. The main outcomes included pain, knee function, stiffness, WOMAC (total), physical function, arthritis self-efficacy (ASE-pain), arthritis self-efficacy (ASE-other symptoms), mental health, and quality of life.

**Results:**

Thirteen randomized controlled trials (RCTs) were finally included (*n* = 1610). Meta-analysis showed differences in pain, knee function, stiffness, ASE-pain, ASE-other symptoms, mental health, and quality of life between the self-management and control groups. Of the nine outcomes evaluated, four were highly heterogeneous, and the quality of evidence ranged from very low to moderate.

**Conclusion:**

The meta-analysis results showed that self-management might help improve the pain, knee function, stiffness, ASE, mental health, and quality of life in patients with KOA. However, it has no significant effect on WOMAC (total) and physical function. Considering that this study has some limitations, we cannot draw clear conclusions based on the results of this study. Nevertheless, we offer much needed insight and encourage more rigorously designed and implemented RCTs in the future to substantiate our conclusions.

## 1. Introduction

Osteoarthritis is the most common chronic joint disease and one of the main causes of pain and disability, while knee osteoarthritis (KOA) is the most common joint disease [1, 2]. It is estimated that the prevalence of KOA in men aged over 60 years is 5% to 15%, while it is as high as 10% to 25% in women of the same age group [3]. KOA can lead to joint pain, muscle weakness, physical disability, and significantly decreased quality of life, while chronic pain can further lead to anxiety, depression, and cognitive dysfunction, severely impacting the daily life of patients [4–7] and increasing the socioeconomic burden [8]. At present, there is no effective treatment for KOA [9]. Relieving pain and preventing the progression of KOA are the main goals of treatment [10]. Although osteoarthritis is incurable, there are many ways to reduce the symptoms of osteoarthritis in affected patients [11, 12]. Arthroplasty is a management strategy for advanced KOA, but it has many potential shortcomings and might not be the best choice for many patients. It is often only considered when other treatment methods are ineffective [13–15], and physicians agree that total knee arthroplasty should not be carried out too early [16]. Oral drugs are the primary choice for patients with KOA. However, long-term use of drugs will also bring about more side effects and result in limited therapeutic effects [17, 18]. Therefore, it is necessary to explore more effective nonsurgical and nonpharmacological treatment options for KOA.

According to the International Association for the Study of Pain (IASP), the pain symptoms of chronic diseases can be managed but cannot be cured [19]. Therefore, it is imperative to manage the disease with safe, effective, and low-cost treatments [20]. As a chronic joint disease, osteoarthritis is related to patients' daily habits, behavior, and lifestyle [21]. Self-management is considered an effective strategy for treating chronic diseases, including the treatment of osteoarthritis [22]. Self-management refers to the ability of patients to manage diseases, symptoms, and treatments, as well as lifestyle, and mental and physical changes caused by diseases [23, 24]. Unfortunately, many patients tend to pay little attention to the management and control of diseases, lacking the ability to self-manage, and cannot make the right decisions related to health [25]. Therefore, it is important to sensitize patients on the importance of disease management. According to the Osteoarthritis Research Society International (OARSI) (2014) recommendations, nonpharmacological therapies, which include self-management, are central to the treatment of KOA [17]. Other clinical guidelines for osteoarthritis also advocate self-management as a nonpharmacological first-line treatment [26]. Clinicians are becoming increasingly interested in the role of self-management intervention in the treatment of KOA, and recently more clinical trials have been conducted. Previous literature reports that self-management interventions are crucial in disease prevention and control as well as health promotion [27]. Published studies have found that self-management intervention can improve pain, joint function, physical function, and quality of life in patients with KOA [28–31]. However, some studies have found that self-management intervention does not significantly improve patients' pain, joint function, and self-efficacy to manage KOA [32, 33]. In addition, other clinical studies have also produced conflicting research results [34, 35]. Given the widespread use of self-management in KOA and the increasing interest in self-management among clinicians, it is important to explore and further clarify the effects of self-management in KOA.

In recent years, increasing RCTs have been conducted on KOA self-management, exploring and analyzing the efficacy of this intervention strategy. However, these published RCTs have produced widely varying conclusions, and the evidence is insufficient. It is difficult for clinicians to benefit from these clinical studies. Hence, the need to conduct a systematic review and meta-analysis based on the latest published RCTs to evaluate their efficacy. To our knowledge, the role of self-management in patients with KAO has not been systematically reviewed. A systematic review with a meta-analysis can help clarify whether self-management is clinically beneficial for KOA and which outcomes are improved. It has some guidance and reference significance for the clinical application of self-management in the treatment of KOA. Clinicians and patients with KOA may benefit from this study. Herein, we aim to systematically evaluate the effectiveness of self-management for KOA and summarize the latest evidence of the clinical efficacy of self-management on KOA based on the published literature.

## 2. Methods

### 2.1. Ethics and Approval

This systematic review and meta-analysis protocols have been registered in PROSPERO (https://www.crd.York.ac.uk/prospero/;%20ID:%20CRD42021259338). This systematic review and meta-analysis were carried out in accordance with the recommendations of PRISMA [36]. All the extracted data are based on published RCTs and do not require ethical approval.

### 2.2. Search Strategies

We searched MEDLINE (through PubMed), EMBASE, Cochrane library, and Web of Science databases until September 17, 2021. The following search terms were used: “osteoarthritis, knee,” Knee osteoarthritis,” Knee Osteoarthritides,” Osteoarthritis of Knee,” Osteoarthritis of the knee,” Self-Management,”“ Self-Management,” Management, Self,” Randomized Controlled Trial,” Clinical Trial,” random allocation.” The specific search strategy is shown in the Supplementary Appendix. The first researcher searched the MEDLINE database, the second researcher searched EMBASE and Cochrane Library databases, the third researcher searched the Web of Science database, and the fourth researcher repeated the search process and search results of all databases. In addition, there was no error in the retrieval process and retrieval results, and the four researchers reached a consensus on the final retrieval results.

### 2.3. Eligibility Criteria

#### 2.3.1. Patients

Included participants were diagnosed with KOA based on the criteria of the American College of Rheumatology (ACR) [37] or by a physician based on the clinical and radiographic features of the patient. There were no restrictions on participants' age, duration of disease, the severity of disease, etc. Participants who have previously undergone total knee arthroplasty will not be included.

#### 2.3.2. Interventions

Self-management refers to patients' participation in activities to promote physical health and prevent disease-related adverse events, including the patients' ability to manage diseases and physical and psychological changes caused by lifestyle, as well as their ability to manage symptoms and treat diseases [19, 23, 24, 38]. The study included a structured self-management program. The main components of self-management may include developing the management skills of osteoarthritis, such as providing patients with osteoarthritis education and knowledge, strengthening the interaction between doctors and patients, and then promoting and stimulating patients' ability to manage osteoarthritis and deal with diseases, and setting relevant goals and formulating action plans. Studies that provided only educational information or focused on psychotherapy interventions were excluded.

#### 2.3.3. Comparisons

Any type of control group could be included in this study, such as routine care, standard treatment, and spa therapy. Routine care means that doctors or nurses provide patients with some general knowledge and information about KOA and some suggestions for relieving pain, as well as some paper materials about KOA for patients to read by themselves. Standard treatment means that patients enrolled in the study continue to receive the usual treatment for KOA prescribed by their doctors. Spa therapy refers to bathing in natural mineral water with a temperature of more than 20 degrees Celsius and rich in minerals for about 20 to 30 minutes.

#### 2.3.4. Outcomes

For inclusion in this review, RCTs had to meet at least one of the following primary outcomes or secondary outcomes:

Primary outcomes:

1. Pain refers to a kind of unpleasant subjective feeling and emotional experience caused by various injurious stimuli acting on the body and causing tissue damage. Pain was measured using the visual analog scale (VAS), Knee Injury and Osteoarthritis Outcome Score (KOOS), or Western Ontario and McMasters University osteoarthritis index (WOMAC).
2. The Knee function refers to the functional state and limitation degree of the Knee joint during various activities. Knee function was measured using the Knee Injury and Osteoarthritis Outcome Score (KOOS) or Western Ontario and McMasters University osteoarthritis index (WOMAC).
3. Stiffness refers to varying degrees of knee dysfunction, including reduced range of motion and rigidity of the joint. Stiffness was measured using the Western Ontario and McMasters University osteoarthritis index (WOMAC).
4. WOMAC (total) is the total score of the WOMAC score, which evaluates the overall functional status of the knee joint. The score includes pain, stiffness, and joint function.
5. Physical function is an important factor of health evaluation, which refers to various body systems' physiological and psychological functional states. Physical function was measured using the Health Assessment Questionnaire (HAQ), The MOS 36-item Short-Form Health Survey (SF-36), or The MOS 36-item Short-Form Health Survey Taiwan Version (T–SF–36).
6. ASE refers to a patient with osteoarthritis prediction and judgment about his or her ability to perform a certain action or a patient with osteoarthritis confidence process about whether he or she can use his or her skills to perform a certain action. ASE was measured using the Arthritis Self-Efficacy scale (ASE).
7. Mental health refers to the patient's psychological state in all aspects, including the comprehensive evaluation of the patient's mood, emotion, attitude, cognition, and other aspects. Mental health was measured using The MOS 36-item Short-Form Health Survey (SF-36), The MOS 36-item Short-Form Health Survey Taiwan Version (T–SF–36), or Hospital Anxiety and Depression (HAD).
8. Quality of life refers to the quality of life related to the patient's health, including a comprehensive assessment of the patient's physical health, mental health, and social status. Quality of life was measured using The MOS 36-item Short-Form Health Survey (SF-36) or Knee Injury and Osteoarthritis Outcome Score (KOOS).

#### 2.3.5. Studies Types

Only RCTs were included; the language of literature was restricted to those published in English.

### 2.4. Exclusion Criteria

Excluded literature involved those that used forms that could not be merged with the data, such as the use of pictures, or the median (quartile) for statistical results, or other forms that could not be merged. In this case, if the data cannot be obtained by contacting the author, the literature was excluded.

### 2.5. Data Extraction

We used Microsoft Excel (2016) software to extract the data required for the study. Before data extraction, two researchers created data extraction forms to independently extract the information needed for the study from each literature. The extracted data mainly included author, publication year, country, study types, sample size, sex ratio, number of dropouts, participant characteristics (age, diagnostic criteria, and symptom duration), interventions, outcomes, and follow-up time. The extracted data were the results of follow-up measurements. After independent data extraction, the two researchers cross-checked each other's extracted data. If there is any doubt about the data, the authors will be contacted for further confirmation. There was no dispute during data extraction, and the two researchers reached a consensus on the extracted data.

### 2.6. Risk of Bias Assessment

We used the Risk-of-Bias 2 tool based on Cochrane Handbook Version 6.2, 2021 to assess the risk of bias in all the included literature. The risk of bias assessed by the Risk-of-Bias 2 tool includes five domains, including:

1. the randomization process
2. deviations from the intended interventions
3. missing outcome data,
4. measurement of the outcome, and
5. selection of the reported result.

Each domain can evaluate low risk, high risk, and some concerns based on the algorithm and then an overall judgment based on the criteria was made (low risk, high risk, and some concerns). Two independent researchers assessed the risk of bias, and a third researcher resolved controversial assessments.

### 2.7. Rating Quality of Evidence

We used the Grading of Recommendations, Assessment, Development and Evaluation (GRADE) system to assess the level of evidence for each outcome. The assessment was carried out in accordance with the GRADE guidelines. The level of evidence is divided into high, moderate, low, and very low.

### 2.8. Statistical Analysis

All the data were merged, and the images were produced using Review Manager V 5.4 (Cochrane Collaboration, Oxford, England) and the Stata software V 16.0 (Stata Corporation, College Station, TX, USA). The forest plot was used to display the results of each merge visually. The measurement outcomes included in this study are all continuous variables. Standard mean differences (SMD) or mean differences (MD) were used for the pooled data, and 95% confidence intervals (CIs) were also calculated. The *I*^*2*^ test was used to assess the heterogeneity between studies. *I*^*2*^ > 50% is considered to have significant heterogeneity [39]. When there is significant heterogeneity, the random-effects model was used; when there is no significant heterogeneity, the fixed-effects model was used. When evaluating the same outcome using different interventions, we performed subgroup analyses based on different interventions. When the interventions in the control group were inconsistent, subgroup analysis was conducted according to different interventions; only the results of subgroup analysis were retained, and the results of different subgroups were not pooled. We used the funnel plot and Trim and Fill Method to assess publication bias.

## 3. Results

### 3.1. Study Selection

**Figure 1:**
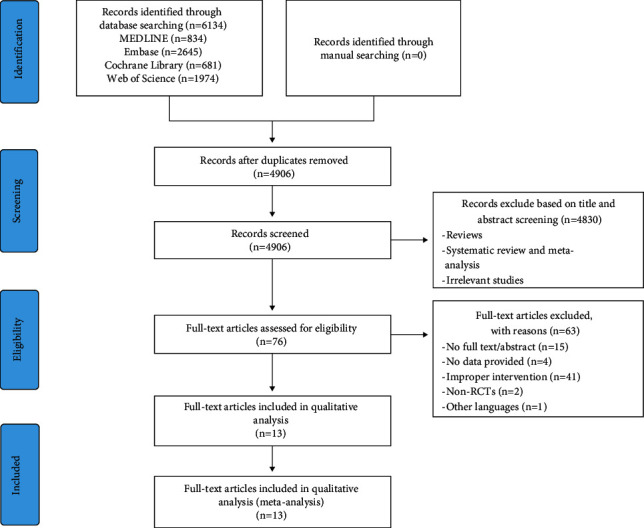
Flowchart of study selection.

We initially searched 6134 related studies from four electronic databases (MEDLINE, Embase, Cochrane Library, and Web of Science) and imported them into NoteExpress 3.3.0 software to screen for duplicate literature, titles, and abstracts. 1228 repeated studies were excluded, and 4830 studies were excluded through reading titles and abstracts. 76 studies were downloaded in full to further determine whether they met the inclusion criteria. Ultimately, thirteen studies remained. After further reading, it was determined that they met the inclusion criteria and were included in this meta-analysis [28, 31–33, 40–48] (Figure 1).

### 3.2. Study Characteristics

#### 3.2.1. Overview of the Included Studies

Table 1 shows the characteristics of these 13 studies, with all included studies being RCTs. The included studies were published from 2004 to 2021. These studies involved seven countries: China [33, 46–48], Thailand [31], Australia [28], Iran [40–42, 44], France [32], USA [43], and Brazil [45]. A total of 1610 participants were included in the 13 RCTs, 853 in the self-management group and 757 in the control group. The 1,610 participants came from different countries, including 607 from China, 40 from Thailand, 136 from Australia, 344 from Iran, 106 from France, 186 from the United States, and 191 from Brazil. Six studies were based on the diagnosis of KOA based on clinical and radiographic features by a physician [28, 31, 40–42, 44], while the remaining seven studies were based on the diagnosis criteria of the American College of Rheumatology [32, 33, 43, 45–47]. The sample size of RCTs ranged from 40 to 205, and the average age of participants ranged from 52.35 to 79.26. Twelve studies reported the dropout rates, ranging from 0 to 52%.

#### 3.2.2. Intervention Characteristics and Outcome Measures

Table 2 shows the characteristics of interventions in these 13 RCTs, including the type of intervention, duration of intervention, outcomes, and follow-up duration. To compare interventions between the self-management group and the control group, three studies used self-management + routine care vs. routine care [31, 43, 45], six studies used self-management vs. routine care [28, 33, 40, 41, 44, 46], three studies used self-management + standard treatment vs. standard treatment [42,47,48], and one study used self-management + spa therapy vs. spa therapy [32]. The included studies used different outcomes. The outcomes of these 13 RCTs include pain, knee function, stiffness, WOMAC (total), physical function, ASE, mental health, and quality of life. Ten studies that assessed pain used VAS scores [33, 40, 41, 44, 47, 48], WOMAC scores [28, 43, 45], and KOOS scores [42], respectively. Two studies assessed stiffness using WOMAC scores [28, 45]. Five studies assessed knee function using WOMAC scores [28, 32, 43, 45] and KOOS scores [42], respectively. Five studies assessed physical function using HAQ [41, 47, 48], SF-36 [28] and T–SF–36 [33], respectively. Four studies assessed arthritis self-efficacy using the ASE scale [32, 46–48]. Three studies assessed mental health using SF-36 [28], T–SF–36 [33], and HAD [32], respectively. Two studies assessed the quality of life using SF-36 [31] and KOOS [42], respectively.

### 3.3. Self-Management Program Components

The main components of the self-management program included the following: patient education, goal-setting and action planning, exercise components, diet or weight management, pain management, medication, motivation, peer support, patient-therapist communication, and related lifestyle management (such as sleep, exercise type, emotions) [49]. All studies included education (*n* = 13) and exercise components (*n* = 13) as components of self-management. Pain management (*n* = 10), related lifestyle management (*n* = 10), patient-therapist communication (*n* = 9), and motivation (*n* = 9) were also frequently used in self-management. In addition, other self-management components included goal-setting (*n* = 7), action planning (*n* = 8), diet or weight management (*n* = 7), medication (*n* = 7), and peer support (*n* = 2). Some theories were applied in the intervention of self-management, such as The Individual and Family self-management Theory (IFSMT) (*n* = 1), Social Cognitive Theory (SCT) (*n* = 1), and Self-Efficacy Theory (SET) (*n* = 4) (Table 3).

### 3.4. Risk of Bias

**Figure 2:**
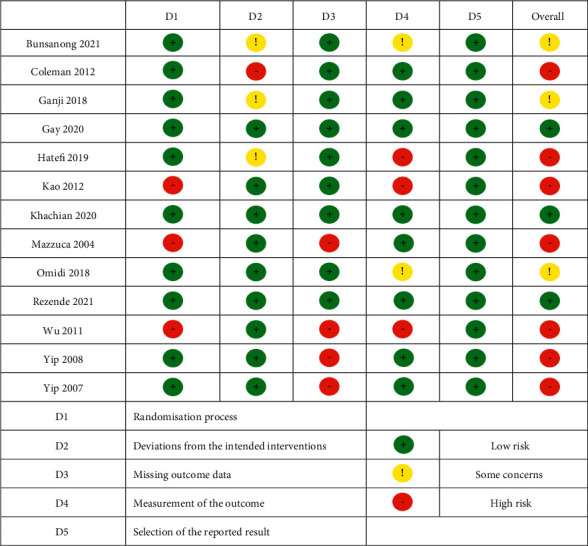
Risk of bias graph.

Figure 2 shows the risk of bias based on the 13 RCTs assessed by the Risk-of-Bias 2 tool. The randomization process of three studies was rated as high risk [33,43,46], and the randomization process of the other ten studies was rated as low risk [28, 31, 32, 40–42, 44,45, 47, 48]. For one study, deviations from the Intended interventions were rated as high risk [28], For nine studies, the Deviations from the Intended interventions were rated as low risk [32, 33, 41–48], and for three studies, the Deviations from the Intended Interventions was rated as some concerns [31, 40, 41]. The missing outcome data of four studies were rated as high risk [43, 46–48], and the missing outcome data of the other nine studies were rated as low risk [28, 31–33, 40–42, 44, 45]. Measurement of the outcome was rated as high risk [33, 41, 46] in three studies, and there were some concerns [31, 44] in two studies. The outcome measurement in the other eight studies was rated as low risk [28, 32, 40, 42, 43, 45, 47, 48]. The selection of the reported results of all studies was rated as low risk. According to the evaluation results of each domain, the overall bias risk of each literature was finally evaluated, of which seven were rated as high risk [28, 33, 41, 43, 46–48], three were rated as some concerns [31, 40, 44], and the other three were rated as low risk [32, 42, 45].

### 3.5. Quality of Evidence

For each measurement outcome, the GRADE system was used to assess the level of evidence. The level of evidence of pain, knee function, ASE (Pain), and ASE (other symptoms) were rated as moderate. Physical function and mental health were rated as low. The level of evidence of stiffness, WOMAC (total), and quality of life were rated as very low (Table 4).

### 3.6. Synthetic Results

#### 3.6.1. Pain

**Figure 3:**
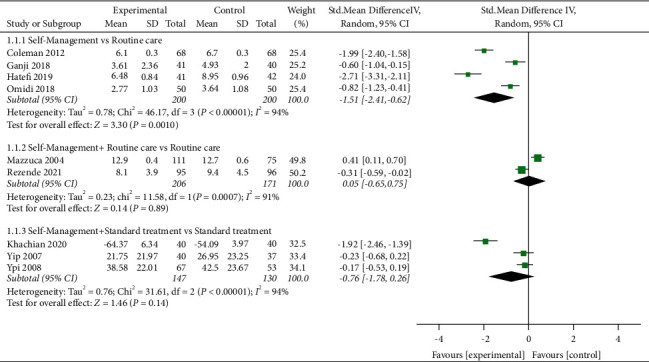
Meta-analysis on pain.

Ten studies evaluated pain and included 1160 participants. Six studies assessed pain with VAS [32, 40, 41, 44, 47, 48], three studies assessed pain with WOMAC score [28, 43, 45], and one study assessed pain with KOOS [42]. The lower the VAS score and WOMAC score, the less painful, and the higher the KOOS score, the less painful. Of the ten RCTs, six studies reported that self-management improved pain in patients with KOA (*P* < 0.05) [28, 40–42, 44, 45], while the other four studies reported that self-management did not improve pain in patients with KOA (*P* > 0.05) [32, 43, 47, 48]. One RCT was not included in the meta-analysis, the comparisons involved self-management + spa therapy being compared with spa therapy alone. When self-management + spa therapy was compared with spa therapy alone, the results showed no statistical difference in pain improvement between the self-management group and spa therapy (*P* > 0.05). Nine of the ten RCTs were included in the meta-analysis. When self-management was compared with routine care, the subgroup analysis showed difference in pain improvement in the self-management group [SMD = -1.51, 95% CI (F02D 2.41, F02D 0.62), *I*^*2*^ = 94%, *P*=0.001]. The pooled results showed high heterogeneity, possibly due to differences in pain management among self-management components and different pain scores in the studies. However, when self-management + routine care was compared with routine care alone, the subgroup analysis showed no difference in pain improvement in the self-management group [SMD = 0.05, 95% CI ( F02D 0.65, 0.75), *I*^*2*^ = 91%, *P*=0.89]. The pooled results showed a high degree of heterogeneity, possibly due to data inconsistencies in the studies. Similarly, when self-management + standard treatment was compared with standard treatment alone, subgroup analysis showed no difference in pain improvement in the self-management group [SMD = -0.76, 95% CI ( F02D 1.78, 0.26), *I*^*2*^ = 94%, *P*=0.14]. The pooled results showed high heterogeneity, possibly due to the use of different pain scoring systems (Figure 3).

#### 3.6.2. Knee Function

Knee function was assessed in five studies involving 699 participants. Four studies used the WOMAC score to assess knee function [28, 32, 43, 45], and one study used the KOOS score to assess knee function [42]. The lower the WOMAC score, the better the knee function, while the higher the KOOS score, the better the knee function. Of the five RCTs, three studies reported that self-management improved knee function in patients with KOA (*P* < 0.05) [28, 32, 42], while the other two studies reported that self-management did not improve knee function in patients with KOA (*P* > 0.05) [43, 45]. Three RCTs were not included in the meta-analysis; the comparisons involved

1. self-management was compared with routine care;
2. self-management + spa therapy was compared with spa therapy alone; and
3. self-management + standard treatment was compared with standard treatment.

**Figure 4:**
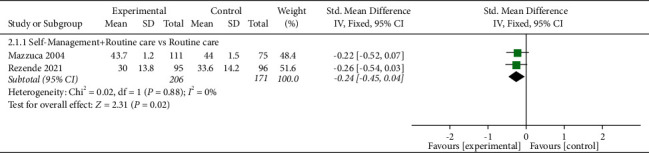
Meta-analysis on Knee function.

When self-management was compared with routine care, the results showed that the self-management group had a significant difference in improved knee function (*P* < 0.05). Similarly, when self-management + spa therapy was compared with spa therapy alone, the results showed that the self-management group also had a difference in improved knee function (*P* < 0.05). In addition, when self-management + standard treatment was compared with standard treatment, the results also showed statistical difference in the self-management group (*P* < 0.05). Two of the five RCTs were included in the meta-analysis. When self-management + routine care was compared with routine care alone, the subgroup analysis showed that there was a significant difference in improved knee function [SMD = -0.24, 95% CI (F02D 0.45, F02D 0.04), *I*^*2*^ = 0%, *P*=0.02] (Figure 4).

#### 3.6.3. Stiffness

Two studies assessed stiffness, involving a total of 327 participants. Both studies used the WOMAC score to assess stiffness [28,45]. The higher the score, the more severe the stiffness. In two RCTs, the comparisons involved

1. self-management was compared with routine care, and
2. self-management + routine care was compared with routine care alone.

When self-management was compared with routine care, the results showed that the self-management group had significantly improved knee stiffness (*P* < 0.05). When self-management + routine care was compared with routine care alone, the results showed that there was no difference in improved stiffness (*P* > 0.05).

### 3.7. WOMAC (total)

**Figure 5:**
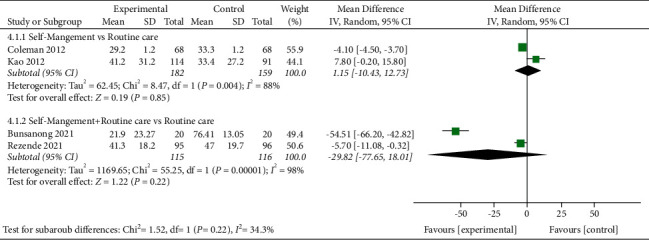
Meta-analysis on WOMAC (total).

WOMAC (total) was evaluated in four studies involving a total of 572 participants. When self-management was compared with routine care, the subgroup analysis revealed that the self-management group showed no difference in improved WOMAC (total) score [MD = 1.15, 95% CI (F02D 10.43, 12.73), *I*^*2*^ = 88%, *P*=0.85]. The pooled results showed a high degree of heterogeneity, possibly due to the large variation in data from one study to another. In addition, when self-management + routine care was compared with routine care alone, the subgroup analysis showed that the self-management group could not statistically improve the WOMAC (total) score [MD = -29.82, 95% CI (F02D 77.65, 18.01), *I*^*2*^ = 98%, *P*=0.22] (Figure 5). The pooled results showed a high degree of heterogeneity, which may be due to the significant differences in data between different studies or to the small number of studies included.

#### 3.7.1. Physical Function

**Figure 6:**
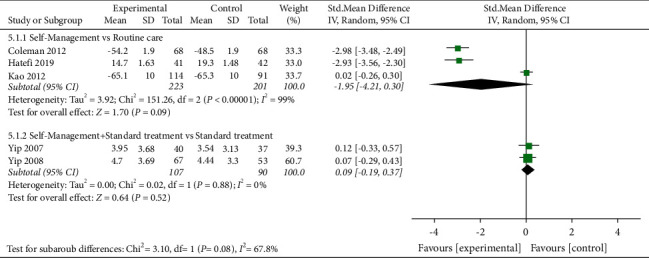
: Meta-analysis on Physical function.

Physical function was evaluated in five studies, and 621 participants were included. Five studies assessed physical function using HAQ [41,47,48], SF-36 [28], or T–SF–36 [33], respectively. The lower the HAQ score, the better the physical function, and the higher the SF-36 and T–SF–36, the better the physical function. When self-management was compared with routine care, the subgroup analysis showed no improvement in the physical function of the self-management group [SMD =  F02D 1.95, 95% CI (F02D 4.21, 0.30), *I*^*2*^ = 99%, *P*=0.09]. The pooled results showed high heterogeneity, which may be caused by data inconsistency in the studies. The results of two of the studies were statistically different, while the results of the other one were not. In addition, when self-management + standard treatment was compared with standard treatment alone, the subgroup analysis also showed that the physical function of the self-management group did not improve [SMD = 0.09, 95% CI (F02D 0.19, 0.37), *I*^*2*^ = 0%, *P*=0.52] (Figure 6).

#### 3.7.2. ASE (Pain)

ASE (Pain) was evaluated in four studies involving a total of 508 participants. Four studies assessed ASE (pain) using the ASE scale [32,46–48]. Of the four RCTs, four studies reported that self-management did not improved ASE (pain) in patients with KOA (*P* > 0.05) [32,46–48]. Two RCTs were not included in the meta-analysis, the comparisons involved

1. self-management + spa therapy was compared with spa therapy alone and
2. self-management was compared with routine care.

**Figure 7:**
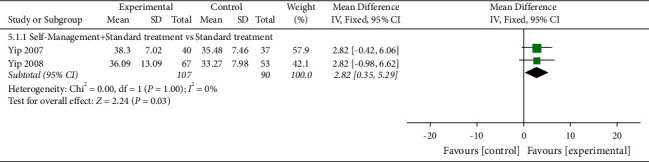
Meta-analysis on ASE (pain).

When self-management + spa therapy was compared with spa therapy alone, the results showed no significant improvement in ASE (pain) (*P* > 0.05). Similarly, when self-management was compared with routine care, the results showed no significant improvement in ASE (pain) in the self-management group (*P* > 0.05). Two of the four RCTs were included in the meta-analysis. When self-management + standard treatment was compared with standard treatment alone, subgroup analysis showed significant improvement in ASE (pain) in the self-management group [MD = 2.82, 95% CI (0.35, 5.29), *I*^*2*^ = 0%, *P*=0.03] (Figure 7).

#### 3.7.3. ASE (Other Symptoms)

Four studies assessed ASE (other symptoms) with a total of 508 participants. Four studies used the ASE scale to evaluate ASE (other symptoms) [32,46–48]. Of the four RCTs, one study reported that self-management improved ASE (other symptoms) in patients with KOA (*P* < 0.05), while the other three studies reported that self-management did not improve ASE (other symptoms) in patients with KOA (*P* > 0.05). Two RCTs were not included in the meta-analysis, the comparisons involved

1. self-management + spa therapy was compared with spa therapy alone and
2. self-management was compared with routine care.

**Figure 8:**
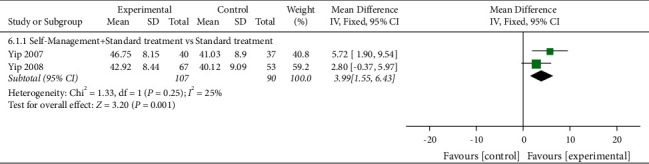
Meta-analysis on ASE (other symptoms).

When self-management + spa therapy was compared to spa therapy, the results showed no significant improvement in ASE (other symptoms) in the self-management group (*P* > 0.05). Similarly, when self-management was compared with routine care, the results showed no significant improvement in ASE (other symptoms) in the self-management group (*P* > 0.05). Two of the four RCTs were included in the meta-analysis. When self-management + standard treatment was compared with standard treatment alone, the subgroup analysis showed a significant improvement in ASE (other symptoms) in the self-management group [SMD = 3.99, 95% CI (1.55, 6.43), *I*^*2*^ = 25%, *P*=0.001] (Figure 8).

#### 3.7.4. Mental Health

**Figure 9:**
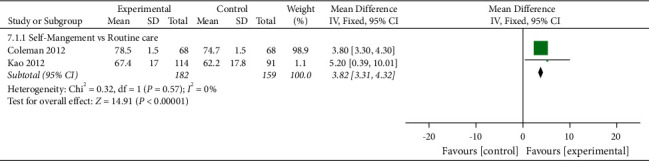
Meta-analysis on Mental health.

Mental health was assessed in three studies involving a total of 447 participants. Three studies assessed mental health using SF-36 [28], T–SF–36 [33], or HAD [32], respectively. The higher the SF-36 and T–SF–36 scores, the better the mental health, and the lower the HAD scores, the better the mental health. Of the three RCTs, three studies reported that self-management improved mental health in patients with KOA (*P* < 0.05) [28, 32, 33]. One RCT was not included in the meta-analysis, and the comparisons involved self-management + spa therapy being compared with spa therapy alone. When self-management + spa therapy was compared with spa therapy alone, the results showed that the mental health of the self-management group was also significantly improved (*P* < 0.05). Two of the three RCTs were included in the meta-analysis. When self-management was compared with routine care, the subgroup analysis showed that the mental health of the self-management group was significantly improved [MD = 3.82, 95% CI (3.31, 4.32), *I*^*2*^ = 0%, *P* < 0.00001] (Figure 9).

#### 3.7.5. Quality of Life

Two studies assessed the quality of life and included a total of 120 participants. Two studies assessed the quality of life using SF-36 [31] or KOOS [42], respectively. The higher these scores are, the better the quality of life. In two RCTs, the comparisons involved

1. self-management + routine care was compared with routine care alone and
2. self-management + standard treatment was compared with standard treatment alone.

When self-management + routine care was compared with routine care alone, the results showed that there was difference in improved quality of life (*P* < 0.05). In addition, when self-management + standard treatment was compared with standard treatment alone, the results showed that the quality of life of the self-management group was also significantly improved (*P* < 0.05).

#### 3.7.6. Publication Bias

**Figure 10:**
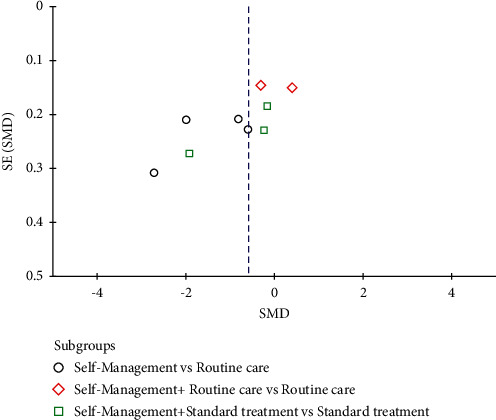
Funnel plot.

**Figure 11:**
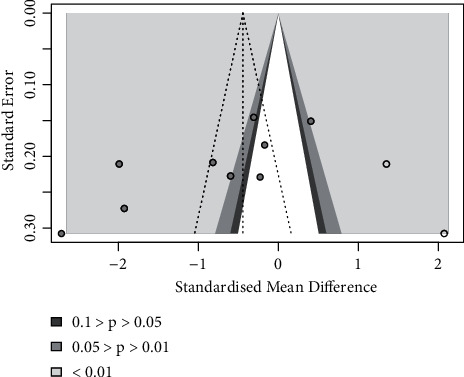
Trim and fill method.

When more than ten studies were included in the meta-analysis, the publication bias of these studies should be evaluated [50]. In this meta-analysis, a total of nine outcomes were evaluated, and only pain was evaluated by ten studies. However, of the 10 studies that assessed pain, only 9 were included for meta-analysis. We made a funnel that showed asymmetry, suggesting that there may be publication bias (Figure 10). Thus, we used the Trim and Fill Method to make an assessment, revealing that the missing data were distributed in statistically significant areas, suggesting that there was no publication bias (Figure 11).

## 4. Discussion

This study aims to systematically evaluate the effectiveness of self-management for KOA. The main evaluation outcomes include pain, knee function, stiffness, WOMAC (total), physical function, ASE, quality of life, and mental health. This study showed that the self-management program might improve pain, knee function, stiffness, ASE, mental health, and quality of life in patients with KOA compared to the control group. However, it has no significant influence on WOMAC (total) and physical function. In this study, a total of nine outcomes were assessed, including four highly heterogeneous outcomes, including pain, knee function, WOMAC (total) score, and physical function. With regard to the heterogeneity of pain, some studies used pain management in self-management, and some did not, possibly due to differences in interventions, and there were also some variations in the use of medications. Different studies used different pain scores, and the evaluation of different pain scores differed greatly, so did the data obtained, which may greatly influence the heterogeneity of pain. The high heterogeneity of knee function may be due to interventions and was significantly reduced following subgroup analysis of different interventions. The high heterogeneity of the WOMAC (total) score may be due to the large variation in results between studies and the small number of studies included. The high heterogeneity of physical function may be caused by the intervention measures used. After the subgroup analysis of these intervention measures, the heterogeneity of one subgroup was significantly reduced. In addition, in this study, the interventions in the control group were not uniform. Since the interventions in the control group were not uniform, and the heterogeneity was high after data were pooled, we conducted subgroup analysis according to different interventions but did not pool the data between different subgroups. For this reason, we cannot draw a definite conclusion at present. The overall effect of self-management intervention on KOA is still unclear, and more studies are needed to verify our findings in the future.

The conclusions of this study support the Individual and Family Self-Management Theory (IFSMT) view that the use of knowledge, beliefs, and self-management skills by patients and their families can help improve the curative effect on the patient's health and quality of life [51]. Although a previous meta-analysis [49] reported the digital self-management of osteoarthritis, that study included eight randomized controlled trials, two studies of which involved intervention in patients with KOA, while the remaining six studies included intervention in patients with both knee osteoarthritis and hip osteoarthritis or other types of osteoarthritis. Knee osteoarthritis and hip osteoarthritis are different in many ways, and the results of incorporating a mix of osteoarthritis patients can vary widely. This is why our study included 13 randomized controlled trials, all of which used self-management to intervene in KOA only. Brand et al. [52] conducted a meta-analysis on the efficacy of self-management therapy combined with exercise therapy in the treatment of KOA and analyzed the efficacy of self-management combined with exercise therapy and self-management alone in the treatment of KOA. They found that both interventions achieved small to moderate effects regardless of whether exercise therapy was included. Consistent with our findings, the study also found that self-efficacy plays an important role in self-management interventions. Chodosh et al. [22] conducted a meta-analysis on the effectiveness of self-management in the treatment of chronic diseases, including patients with hypertension, diabetes, and osteoarthritis. They found that the improvement of pain and joint function by self-management was statistically different, but there seemed to be no clinical benefit. However, that study did not reveal the types of osteoarthritis in the patients enrolled, and only pain and joint function outcomes were assessed. In this study, only patients with KOA were included, and nine outcomes were evaluated, which offer a more significant reference to reflect the effectiveness of self-management in the treatment of KOA. Kroon et al. [53] also performed a meta-analysis on the effectiveness of self-management on osteoarthritis, but they included inconsistent interventions in the control group, which included attention control and routine care; they found that compared with attentional control, self-management might not improve pain, function, and quality of life in patients with osteoarthritis, but may improve when compared with conventional care. However, the study also included patients with multiple types of osteoarthritis, including KOA, hip osteoarthritis, and arthritis of the hands, which could ultimately affect the results. The pathogenesis and treatment of different osteoarthritis are different, and the therapeutic effect is also different. When evaluating the efficacy of an intervention, the inclusion of only one subtype of the same disease contributes to a better evaluation, and the conclusions can provide better reference and guidance for clinical use. Therefore, only patients with KOA were included in our study.

The purpose of self-management is to allow patients to make necessary behavior changes to improve their health [28]. Unlike the traditional medical model, self-management intervention focuses on the interaction and coordination between patients and medical workers, rather than the one-way passive management and care provided by the medical staff [24]. The self-management model is advocated to strengthen the daily management of disease through the patient's responsibility [54]. Although self-management overlaps with education, psychology, and rehabilitation, it also focuses on self-efficacy construction, self-monitoring, and self-adaptation, setting goals and action plans, making decisions and solving problems, as well as the interaction between patients and healthcare workers; these advantages distinguish self-management from other intervention models such as patient education [22, 54–58]. Theoretically, self-management includes the patient's management of illness and symptoms, daily behavior management, emotion management, role management, health-related decision-making, problem-solving ability, and taking relevant actions [24, 59, 60]. Two important theories explain self-management, including self-efficacy theory and social cognition theory [61, 62]. Self-efficacy is an essential part of self-management and is closely related to self-management [28]. Self-efficacy can be defined as people's belief in their ability to maintain their specific behaviors; many studies believe that self-efficacy significantly impacts patients' ability to manage chronic diseases [63, 64]. Self-management interventions focus on promoting patients' self-efficacy construction, enhancing patients' perception of disease control by improving self-efficacy [58, 65]. Previous studies have found that self-efficacy is closely related to physical health, and improving self-efficacy can improve patients' physical health, including pain, mental health, and physical function [66, 67]. For chronic diseases, the measurement results of self-management mainly include pain, physical function, quality of life, mental health, self-efficacy, etc.; There is no specific way to measure the effectiveness of self-management, and clinical practice guidelines do not recommend using any specific tool to measure the effectiveness of self-management [68, 69].

Although the mechanism through which self-management is effective in KOA remains poorly understood, there seems to be a consensus among investigators that self-management increases patient compliance with various interventions, including pharmacological and other interventions [22, 70]. KOA is closely related to the patient's daily behaviors, habits, and lifestyle, and by adjusting the correct lifestyle and habits, the risk factors of KOA and other complications can be reduced, thus lowering the incidence of KOA [28, 71, 72]. Published literature has found that self-management can protect physical function and improve self-efficacy in patients with KOA, thereby reducing pain and improving their quality of life [28, 35]. In addition, self-management can improve patients' awareness of disease and health and their ability to manage disease-related symptoms [73], as well as boost patients' confidence in managing disease and symptoms, which is an important factor for patients to change their behavior and can also have an impact on patient's health status [74]. Our results show that the self-management strategy significantly improved pain, knee function, stiffness, ASE, mental health, and quality of life in patients with KOA. This study reported consistent results with a meta-analysis conducted by Park et al. [75]. Although this study found no significant improvement in WOMAC (total) and physical function, possibly due to the small sample size or publication bias in some studies, the pooled results showed a trend toward improvement.

In the context of the global COVID-19 pandemic, there are some barriers to providing face-to-face health care services to patients, with face-to-face health care services between doctors and patients significantly reduced. It is especially important to provide some medical technical support to patients through other methods. Although some progress has been made in the treatment of KOA, self-management interventions are mainly based on patient self-feedback, and there is a lack of standardized monitoring of patients outside the clinical environment. Wearable technology has important implications for the self-management of patients with KOA in real life [76].Wearable technology can realize information sharing between doctors and patients, strengthen patient monitoring and communication between doctors and patients, and make the clinical application of self-management more standardized. Wearable technology using artificial intelligence may promote the development of personalized medicine [76, 77]. In addition, a review systematically evaluated the clinical efficacy of wearable technology for patients after knee arthroplasty [78], and the application of wearable technology in early postoperative rehabilitation is also of great significance. In view of the application of wearable technology in patients with KOA and its contribution to the self-management of patients with KOA, future clinical self-management interventions should be combined with wearable technology to promote the integration of self-management and wearable technology to make the application of self-management in nonclinical environments more standardized.

Although we have strictly screened the included literature, there are still some limitations in the included literature, which may have a potential impact on the results of the study. Firstly, not all studies have detailed the randomization methods, allocation concealment, implementation bias, and detection bias, which reduce the quality of the literature. Secondly, the sample size of the literature included in this study is small, affecting the comparison between groups. Although the small sample size can be used for Meta-analysis, the conclusions are preliminary, and caution should be warranted when interpreting them [79]. Thirdly, the number of dropouts in some studies is large, and the results may be biased. Fourthly, the included literature had follow-ups, but the duration was inconsistent, and the difference in follow-up time was significant. Therefore, the subgroup analysis could not be carried out and the pooled results may have significant heterogeneity. Thus, we extracted data from the last follow-up time of each study to assess the long-term efficacy of self-management. However, such pooled data may be affected by other factors, and the conclusions drawn are not clear. In addition, in our included literature, the length of intervention time was inconsistent. However, due to the small number of included literature, we could not conduct subgroup analysis for different intervention times, potentially affecting our pooled results. Finally, most of the studies included did not report the Kellgren–Lawrence (KL) grade of KOA, so we could not evaluate the efficacy of self-management for various degrees of KOA. Given some limitations of the studies we included, the conclusions of this study are preliminary and we cannot draw definitive conclusions. At the same time, more caution should be exercised when interpreting the clinical efficacy of self-management for KOA.

Although we had completed the protocol registration in PROSPERO before the study started, due to literature limitations, there are some differences between our manuscript and the pre-registered protocol. First, the pre-registered protocol included three outcomes: fatigue, exercise time, and the number of drug consultations. However, we did not report the three results because there was only one study report for each of the three results, so meta-analysis could not be conducted, and the number of studies was too small for reference significance. Second, four Chinese databases were included in the pre-registered protocol. However, due to the low quality of the literature, after discussion by the research team, we limited the publication language to English and excluded 4 Chinese databases. Third, the retrieval time of the pre-registered protocol is until May 30, 2021. Due to the busy work schedule, the retrieval time is extended until September 17, 2021. Fourth, we performed subgroup analyses due to inconsistent interventions in the control group, but data between the different subgroups were not pooled. Although there are some differences between the work done in our manuscript and the pre-registered protocol, overall, this study was conducted according to the pre-registered protocol.

### 4.1. Limitations

Although the included literature was rigorously screened, this study still has some limitations. Firstly, the study included only studies published in English, which may have some bias. Secondly, although we have developed search strategies before retrieval, different databases are searched by different researchers, so there may be some bias. Thirdly, we screened a large number of irrelevant literature by reading titles and abstracts, and there was the possibility of missing some important literature. Fourthly, the study limited the intervention to self-management vs. other therapies or self-management + other therapies vs. other therapies. This mix of interventions could potentially impact the study results, and the conclusions obtained were not clear. In addition, the interventions in the control group were not consistent, and we did not pool the different subgroups, so we could not assess the overall effect of self-management. Finally, due to methodological differences in the included literature, the level of evidence presented in this study is low. Therefore, the conclusions drawn from this study are preliminary, and further verification by more high-quality and large sample RCTs are warranted.

### 4.2. Implications for Further Research and Practice

We acknowledge that there are significant difficulties in conducting this type of randomized controlled trial, but there are ways to improve the limitations of the current literature. First, future RCTs should strictly follow the guidelines of CONSORT [80], and studies should report specific randomization methods and allocation concealment, while strict blinding methods should be implemented. Second, future studies should extend the duration and frequency of follow-up to evaluate the efficacy at different time points, which is of great significance for clinical implementation. Third, participants should be classified by the severity of methods used to treat KOA, such as the KL grade and course of the disease, and the efficacy of interventions for different degrees of KOA may vary. Fourth, future studies should include more samples and reduce the dropout rate. Large-sample RCTs can increase the reliability of conclusions. Finally, despite these limitations in the included studies, since self-management is a nonpharmacological intervention with no side effects and low economic cost, more and more RCTs on self-management should be conducted in the future.

## 5. Conclusion

The meta-analysis results showed that self-management might help improve the pain, knee function, stiffness, ASE, mental health, and quality of life in patients with KOA. However, it has no significant effect on WOMAC (total) and physical function. Considering that this study has some limitations, we cannot draw clear conclusions based on the results of this study. Nevertheless, we offer much needed insight and encourage more rigorously designed and implemented RCTs in the future to substantiate our conclusions.

## Figures and Tables

**Table 1:** Study characteristics.

References	Diagnosis criteria	Country	Study types	Mean age (SD), years		Sample size		Male/Female		Symptom duration in years (SD)		Dropout rates (%)	
				SMG	CG	SMG	CG	SMG	CG	SMG	CG	SMG	CG
Bunsanong et al. [31]	Physician diagnosed	Thailand	RCT	52.35 (5.82)	63.00 (5.47)	20	20	Not reported		3.20 (3.41)	3.30 (2.54)	5.0	15.0
Coleman et al. [28]	Physician diagnosed	Australia	RCT	65.00 (7.90)	65.00 (8.70)	71	75	14/57	23/52	Not reported		4.2	9.3
Ganji et al. [40]	Physician diagnosed	Iran	RCT	65.34 (6.19)	64.58 (4.67)	41	41	Not reported		Not reported		0	2.4
Gay et al. [32]	ACR (1986)	France	RCT	66.60 (6.40)	64.70 (7.10)	54	69	9/45	13/56	12.10 (7.70)	11.20 (7.70)	3.7	21.7
Hatefi et al. [41]	Physician diagnosed	Iran	RCT	75.36 (6.58)	79.26 (14.17)	41	42	0/41	0/42	4.43 (1.00)	4.02 (1.17)	0	0
Kao et al. [33]	ACR (1991)	China	RCT	67.30 (10.10)	68.20 (11.20)	114	91	22/92	26/65	Not reported		17.5	37.3
Khachian et al. [42]	Physician diagnosed	Iran	RCT	58.97 (Not reported)	58.02 (Not reported)	40	40	12/28	10/30	Not reported		0	0
Mazzuca et al. [43]	ACR (1986)	USA	RCT	61.80 (12.50)	61.80 (11.90)	111	75	29/82	22/53	Not reported		Not reported	
Omidi et al. [44]	Physician diagnosed	Iran	RCT	57.12 (9.16)	58.76 (8.31)	50	50	12/38	10/40	Not reported		8.0	8.0
Rezende et al. [45]	ACR (1991)	Brazil	RCT	63.80 (9.50)	63.20 (8.70)	95	96	16/79	20/76	Not reported		16.8	15.6
Wu et al. [46]	ACR (1986)	China	RCT	67.27 (10.05)	68.18 (11.21)	114	91	22/92	26/65	Not reported		17.5	37.3
Yip et al. [47]	ACR (1991)	China	RCT	64.80 (10.58)	63.40 (10.73)	45	50	5/40	9/41	8.04 (5.92)	6.72 (6.02)	35.5	52.0
Yip et al. [48]	ACR (1991)	China	RCT	65.00 (Not reported)		88	94	Not reported		8.00 (Not reported)		23.8	43.6

ACR, American College of Rheumatology; RCT, Randomized controlled trial; SD, Standard deviation; SMG, Self-mangement group; CG, Control group.

**Table 2:** Intervention characteristics and outcome measures.

References	Type of self-management practice	Intervention characteristics		Main outcomes and results	Follow-up
		Self-management group	Control group		
Bunsanong et al. [31]	Self-management support intervention	Self-management (6 weeks) + routine care	Routine care	1. WOMAC (total); 2. Quality of life (SF36-total);	4 weeks,8 weeks
Coleman et al. [28]	Osteoarthritis of the knee self-management program (OAK)	Self-management (6 weeks)	Routine care	1. Pain (WOMAC); 2. Knee function (WOMAC); 3. Stiffness (WOMAC); 4. WOMAC (total); 5. Physical function (SF36); 6. Mental health (SF36);	8 weeks,24 weeks
Ganji et al. [40]	Self-management program	Self-management (4 weeks)	Routine care	1. Pain (VAS);	4 weeks, 12 weeks
Gay et al. [32]	Self-management exercise program	Self-management (4 weeks) + spa therapy	Spa therapy	1. Pain (VAS); 2. Knee function (WOMAC); 3. ASE (pain); 4. ASE (other symptoms); 5. Mental health (HAD);	12 weeks
Hatefi et al. [41]	Self-management program	Self-management (4 weeks)	Routine care	1. Pain (VAS); 2. Physical function (HAQ);	4 weeks
Kao et al. [33]	Taipei osteoarthritis program (TOAP)	Self-management (4 weeks)	Routine care	1. WOMAC (total); 2. Physical functioning (T-SF36); 3. Mental health (T-SF36);	4 weeks 8 weeks
Khachian et al. [42]	Self-management program	Self-management (6 weeks) + standard treatment	Standard treatment	1. Pain (KOOS); 2. Knee function (KOOS); 3. Quality of life (KOOS);	8 weeks
Mazzuca et al. [43]	Osteoarthritis Care Algorithms	Self-Management (Not reported) + routine care	Routine care	1. Pain (WOMAC); 2. Knee function (WOMAC);	12 weeks 24 weeks 48 weeks
Omidi et al. [44]	Self-management training	Self-management (8 weeks)	Routine care	1. Pain(VAS);	8 weeks
Rezende et al. [45]	OA self-management program	Self-management (8 weeks) + routine care	Routine care	1. Pain (WOMAC); 2. Knee function (WOMAC); 3. Stiffness (WOMAC); 4. WOMAC (total);	24 weeks, 48 weeks, 96 weeks
Wu et al. [46]	Taipei osteoarthritis program (TOAP)	Self-management (4 weeks)	Routine care	1. ASE (pain); 2. ASE (other symptoms);	4 weeks, 12 weeks
Yip et al. [47]	Arthritis self-management programme (ASMP)	Self-management (6 weeks) + standard treatment	Standard treatment	1. Pain (VAS); 2. Physical function (HAQ); 3. ASE (pain); 4. ASE (other symptoms);	7 week, 16 weeks, 48 weeks
Yip et al. [48]	Arthritis self-management programmes (ASMP)	Self-management (16 weeks) + standard treatment	Standard treatment	1. Pain (VAS); 2. Physical function (HAQ); 3. ASE (pain); 4. ASE (other symptoms);	1 week, 16 weeks

OAK, Osteoarthritis of the Knee Self-Management Program; TOAP, Taipei Osteoarthritis Program; ASMP, Arthritis Self-Management Programme; VAS, Visual Analog Scale; WOMAC, Western Ontario and McMasters University Osteoarthritis Index; SF36, The MOS 36-Item Short-Form Health Survey; T-SF36, The MOS 36-Item Short-Form Health Survey Taiwan Version; ASE, Arthritis Self-Efficacy; HAQ, Health Assessment Questionnaire; HAD, Hospital Anxiety and Depression; KOOS, Knee Injury and Osteoarthritis Outcome Score.

**Table 3:** Self-management program components.

References	Education	Goal setting	Action planning	Exercise components	Diet or weight management	Pain management	Medication	Motivation	Peer support	Patient therapist communication	Related lifestyle management	Theory
Bunsanong et al. [31]	✔	✔	✔	✔	NR	✔	✔	✔	NR	NR	NR	IFSMT
Coleman et al. [28]	✔	✔	✔	✔	✔	✔	✔	✔	NR	✔	✔	SCT
Ganji et al. [40]	✔	NR	NR	✔	NR	NR	NR	✔	NR	✔	NR	NR
Gay et al. [32]	✔	✔	✔	✔	NR	NR	NR	NR	NR	✔	NR	NR
Hatefi et al. [41]	✔	✔	NR	✔	✔	✔	✔	✔	NR	✔	✔	NR
Kao et al. [33]	✔	✔	✔	✔	NR	✔	✔	✔	✔	✔	✔	SET
Khachian et al. [42]	✔	NR	✔	✔	✔	✔	✔	✔	NR	✔	✔	NR
Mazzuca et al. [43]	✔	✔	✔	✔	✔	✔	✔	NR	NR	✔	✔	NR
Omidi et al. [44]	✔	NR	NR	✔	✔	✔	NR	NR	NR	NR	✔	NR
Rezende et al. [45]	✔	NR	✔	✔	✔	NR	NR	NR	NR	✔	✔	NR
Wu et al. [46]	✔	✔	✔	✔	NR	✔	✔	✔	✔	✔	✔	SET
Yip et al. [47]	✔	NR	NR	✔	NR	✔	NR	✔	NR	NR	✔	SET
Yip et al. [48]	✔	NR	NR	✔	NR	✔	NR	✔	NR	NR	✔	SET

IFSMT, Individual and Family Self-Management Theory; SCT, Social Cognitive Theory; SET, Self-Efficacy Theory; NR, Not reported.

**Table 4:** Evidence quality rated using the GRADE approach.

Outcomes	No. of studies	Sample size	Risk of bias	Inconsistency	Indirectness	Imprecision	Publication bias	Evidence quality	
Pain	10	1160	Not serious	Serious	Not serious	Not serious	Undetected	⊕⊕⊕ ⊝	Moderate
Knee function	5	699	Not serious	Serious	Not serious	Not serious	Undetected	⊕⊕⊕ ⊝	Moderate
Stiffness	2	327	Serious	Not serious	Not serious	Very serious	Undetected	⊕⊝ ⊝ ⊝	Very low
WOMAC (total)	4	572	Serious	Very serious	Not serious	Not serious	Undetected	⊕⊝ ⊝ ⊝	Very low
Physical function	5	621	Not serious	Very serious	Not serious	Not serious	Undetected	⊕⊕⊝ ⊝	Low
ASE (Pain)	4	508	Serious	Not serious	Not serious	Not serious	Undetected	⊕⊕⊕ ⊝	Moderate
ASE (other symptoms)	4	508	Serious	Not serious	Not serious	Not serious	Undetected	⊕⊕⊕ ⊝	Moderate
Mental health	3	447	Serious	Not serious	Not serious	Serious	Undetected	⊕⊕⊝ ⊝	Low
Quality of life	2	120	Serious	Not serious	Not serious	Very serious	Undetected	⊕⊝ ⊝ ⊝	Very low

ASE, Arthritis Self-Efficacy; WOMAC, Western Ontario and McMasters University osteoarthritis index.

## Data Availability

The data set for this study can be obtained by contacting the corresponding author and will be provided without reservation.
