# Supplementary material for: Self-Management for Knee Osteoarthritis: A Systematic Review and Meta-Analysis of Randomized Controlled Trials
Source: Pain Res Manag. 2022 Mar 2;2022:2681240. doi: 10.1155/2022/2681240 (PMC8906975; doi:10.1155/2022/2681240)
Supplement: Supplementary Materials — Supplementary material can be found in the appendix of this article. The manuscript contains a supplementary material, which is a detailed search strategy for four databases. [file 2681240.f1.zip › 2681240.f1/Search algorithm (1).pdf]

Supplementary Appendix - Search algorithm

Medline(through Pubmed)

#1	*((((osteoarthritis, knee [MeSH Terms]) OR (Knee osteoarthritis [Title/Abstract])) OR (Knee Osteoarthritides [Title/Abstract])) OR (Osteoarthritis of Knee [Title/Abstract])) OR (Osteoarthritis of the Knee [Title/Abstract])*	*27,876*
#2	*((self care[MeSH Terms]) OR (Self care[Title/Abstract])) OR (Self monitoring[Title/Abstract])) OR (group, self help[MeSH Terms])) OR (groups, self help[MeSH Terms])) OR (Self help[Title/Abstract])) OR (Self efficacy[Title/Abstract])) OR (self efficacy[MeSH Terms])) OR (Self administration[Title/Abstract])) OR (Self monitor[Title/Abstract])) OR (self medication[MeSH Terms])) OR (self medications[MeSH Terms])) OR (self regulation[MeSH Terms])) OR (self regulations[MeSH Terms])) OR (Self regulation[Title/Abstract])) OR (Self treatment[Title/Abstract])) OR (Social support[MeSH Terms])) OR (Social support[Title/Abstract])) OR (adaptive behavior[MeSH Terms])) OR (Adaptive behavior[Title/Abstract])) OR (Behavior therapy[Title/Abstract])) OR (behavior therapy[MeSH Terms])) OR (behavior therapy, cognitive[MeSH Terms])) OR (Patient participation[Title/Abstract])) OR (Patient education[Title/Abstract])) OR (patient education[MeSH Terms])) OR (patient education as topic[MeSH Terms])) OR (health education[MeSH Terms])) OR (Health education[Title/Abstract])) OR (Educational therapy[Title/Abstract])) OR (Health communication[Title/Abstract])) OR (Disease management[Title/Abstract])) OR (disease management[MeSH Terms])) OR (Patient Care Management[Title/Abstract])) OR (Management program[Title/Abstract])) OR (Management plan[Title/Abstract])) OR (Self-Management[MeSH Terms])) OR (Self-Management[Title/Abstract])) OR (Self Management[Title/Abstract])) OR (self management[MeSH Terms])) OR (Management, Self[Title/Abstract])*	*785,583*
#3	*#1 AND #2*	*1671*
#4	*(((((((Randomized Controlled Trial [Publication Type]) OR (Clinical Trial [Publication Type])) OR (Randomly [Title/Abstract])) OR (Random [Title/Abstract])) OR (Randomised [Title/Abstract])) OR (Trial [Title/Abstract])) OR (Control [Title/Abstract])) OR (Controlled [Title/Abstract])*	*4,592,050*
#5	*#3 AND #4*	*878*
#6	*Filters: MEDLINE*	*834*

Embase

#1	*'knee osteoarthritis'/exp*	*37,135*
#2	*'knee osteoarthritis':ab, ti*	*19,538*
#3	*'Osteoarthritis of Knee':ab, ti*	*336*
#4	*'Osteoarthritis of the Knee':ab, ti*	*4,235*
#5	*#1 OR #2 OR #3 OR #4*	*40,875*
#6	*'self care'/exp*	*90,340*
#7	*'self care':ab, ti*	*28,407*

#8	*'self management':ab, ti*	*30,356*
#9	*'self management support'/exp*	*21*
#10	*'self monitoring':ab,ti*	*10,810*
#11	*'self monitoring'/exp*	*8,356*
#12	*'self help'/exp*	*14,439*
#13	*'self help':ab,ti*	*8,839*
#14	*'self efficacy':ab,ti*	*38,280*
#15	*'self administration':ab,ti*	*13,991*
#16	*'self medication'/exp*	*11,391*
#17	*'self medication':ab,ti*	*6,133*
#18	*'self regulation':ab,ti*	*10,194*
#19	*'self treatment':ab,ti*	*2,024*
#20	*'social support'/exp*	*99,865*
#21	*'social support':ab,ti*	*53,573*
#22	*'adaptive behavior'/exp*	*61,889*
#23	*'adaptive behavior':ab,ti*	*3,931*
#24	*'behavior therapy'/exp*	*64,462*
#25	*'behavior therapy':ab,ti*	*7,015*
#26	*'patient participation'/exp*	*30,129*
#27	*'patient education'/exp*	*117,786*
#28	*'patient education':ab,ti*	*29,335*
#29	*'health education'/exp*	*350,025*
#30	*'health education':ab,ti*	*40,152*
#31	*'disease management'/exp*	*3,210,069*
#32	*'disease management':ab,ti*	*25,094*
#33	*'management program':ab,ti*	*10,925*
#34	*'educational therapy':ab,ti*	*149*
#35	*'management plan':ab,ti*	*7,539*
#36	*#6 OR #7 OR #8 OR #9 OR #10 OR #11 OR #12 OR #13 OR #14 OR #15 #16 OR #17 OR #18 OR #19 OR #20 OR #21 OR #22 OR #23 OR #24 OR #25 #26 OR #27 OR #28 OR #29 OR #30 OR #31 OR #32 OR #33 OR #34 OR #35 OR #36*	*3,804,934*
#37	*'randomized controlled trial'/exp*	*678,249*
#38	*#5 AND #36 AND #37*	*2,645*

Cochrane Library

#1	*MeSH descriptor: [Osteoarthritis, Knee] explode all trees*	*4,714*
#2	*("knee osteoarthritis"): ti, ab, kw*	*7,751*
#3	*("Osteoarthritis of the Knee"): ti, ab, kw*	*2,120*
#4	*("Osteoarthritis of Knee"): ti, ab, kw*	*525*
#5	*#1 OR #2 OR #3 OR #4*	*10,686*
#6	*MeSH descriptor: [Self-Management] explode all trees*	*550*
#7	*("self-management program"): ti, ab, kw*	*1,279*
#8	*("Self Management"): ti, ab, kw*	*8,806*

#9	*("Self care"):ti,ab,kw*	*11,933*
#10	*("Self monitoring"):ti,ab,kw*	*4,899*
#11	*("self help group"):ti,ab,kw*	*920*
#12	*("Self administration"):ti,ab,kw*	*2,008*
#13	*("Self monitor"):ti,ab,kw*	*4,898*
#14	*MeSH descriptor: [Self Efficacy] explode all trees*	*3,313*
#15	*("Self medication"):ti,ab,kw*	*491*
#16	*MeSH descriptor: [Self-Control] explode all trees*	*312*
#17	*("Self regulation"):ti,ab,kw*	*2,045*
#18	*("Self treatment"):ti,ab,kw*	*232*
#19	*MeSH descriptor: [Social Support] explode all trees*	*3,439*
#20	*("Adaptive behavior"):ti,ab,kw*	*866*
#21	*MeSH descriptor: [Behavior Therapy] explode all trees*	*17,646*
#22	*MeSH descriptor: [Patient Education as Topic] explode all trees*	*9,150*
#23	*MeSH descriptor: [Health Education] in all MeSH products*	*20,741*
#24	*("Educational therapy"):ti,ab,kw*	*95*
#25	*("Patient education"):ti,ab,kw*	*14,318*
#26	*("Health education"):ti,ab,kw*	*11,340*
#27	*MeSH descriptor: [Disease Management] explode all trees*	*5,030*
#28	*("Management program"):ti,ab,kw*	*4,069*
#29	*("Management plan"):ti,ab,kw*	*645*
#30	*#6 OR #7 OR #8 OR #9 OR #10 OR #11 OR #12 OR #13 OR #14 OR #15 OR #16 OR #17 OR #18 OR #19 OR #20 OR #21 OR #22 OR #23 OR #24 OR #25 OR #26 OR #27 OR #28 OR #29*	*63,305*
#31	*#5 AND #30*	*681*

Web of Science

#1	*TS = Knee osteoarthritis*	*67,385*
#2	*TI = Knee osteoarthritis*	*19,028*
#3	*TS = Osteoarthritis of Knee*	*63,064*
#4	*TI = Osteoarthritis of Knee*	*13,925*
#5	*TS = Osteoarthritis of the Knee*	*59,204*
#6	*TI = Osteoarthritis of the Knee*	*8,036*
#7	*TI = KOA*	*218*
#8	*#1 OR #2 OR #3 OR #4 OR #5 OR #6 OR #7*	*67,551*
#9	*TS = (Self Management)*	*171,200*
#10	*TI = (Self Managemen)t*	*14,099*
#11	*AB = (Self Management)*	*83,540*
#12	*TS = (Self-Management)*	*35,594*
#13	*TI = (Self-Management)*	*11,057*
#14	*AB = (Self-Management)*	*23,126*
#15	*TS=(Self care)*	*247,369*
#16	*TI=(Self care)*	*12,663*

#17	TI=(Self monitoring)	5,056
#18	TS=(Self monitoring)	86,560
#19	TS=(Self help)	112,981
#20	TI=(Self help)	3,742
#21	TI=(Self administration)	5,455
#22	TS=(Self administration)	110,323
#23	TS=(Self monitor)	86,560
#24	TI=(Self monitor)	5,056
#25	TI=(Self regulation)	5,637
#26	TS=(Self regulation)	83,505
#27	TS=(Self treatment)	322,687
#28	TI=(Self treatment)	6,652
#29	TI=(Social support)	17,106
#30	TS=(Social support)	339,944
#31	TS=(Behavior therapy)	1,222,962
#32	TI=(Behavior therapy)	6,628
#33	TI=(Patient education)	14,522
#34	TS=(Patient education)	364,869
#35	TS=(Health education)	592,229
#36	TI=(Health education)	25,203
#37	TI=(Educational therapy)	522
#38	TS=(Educational therapy)	59,869
#39	TS=(Disease management)	1,211,615
#40	TI=(Disease management)	35,208
#41	TI=(Management program)	13,800
#42	TS=(Management program)	378,355
#43	#9 OR #10 OR #11 OR #12 OR #13 OR #14 OR #15 OR #16 OR #17 OR #18 OR #19 OR #20 OR #21 OR #22 OR #23 OR #24 OR #25 OR #26 OR #27 OR #28 OR #29 OR #30 OR #31 OR #32 OR #33 OR #34 OR #35 OR #36 OR #37 OR #38 OR #39 OR #40 OR #41 OR #42	4,085,871
#44	TS = Randomized Controlled Trial	625,393
#45	TI = Randomized Controlled Trial	137,466
#46	#44 OR #45	625,393
#47	#8 AND #43 AND #46	1,974
